# Pre-existing comorbidities and hospitalization for COVID-19 are associated with post-COVID conditions in the U.S. veteran population

**DOI:** 10.1038/s43856-025-01057-5

**Published:** 2025-10-24

**Authors:** Aaron J. Miller, Guo Wei, Gregory J. Stoddard, Sujee Jeyapalina, Jayant P. Agarwal

**Affiliations:** 1 Research, George E. Wahlen Department of Veterans Affairs Medical Center, Salt Lake City, UT USA; 2 Division of Plastic and Reconstructive Surgery, Department of Surgery, University of Utah School of Medicine, Salt Lake City, UT USA; 3 Division of Epidemiology, Department of Internal Medicine, University of Utah School of Medicine, Salt Lake City, UT USA

**Keywords:** Viral infection, Epidemiology

## Abstract

**Introduction:**

Although most survivors of COVID-19 return to their baseline health within two weeks, a notable proportion of individuals continue experiencing symptoms, collectively referred to as Post-COVID Conditions (PCC). To better understand risks associated with contracting PCC, this study aimed to determine whether association exists between pre-existing comorbidities, hospitalization for COVID-19 and the subsequent diagnosis of PCC in US veterans.

**Methods:**

This retrospective cohort study collected data from the US Department of Veterans Affairs electronic medical records from September 1, 2021, to July 31, 2023. Participants were limited to those with electronic medical records of a SARS-CoV-2 infection, who received care from the Veterans Affairs hospital system and survived at least 28 days following the infection.

**Results:**

The multivariable logistic regression analysis reveals in hospitalized veterans, chronic obstructive pulmonary disease (COPD) associates with a 21% increase in odds of a PCC diagnosis (adjusted OR 1.21, 95%CI 1.14–1.29; p < 0.001), while in non-hospitalized veterans, chronic kidney disease (OR 1.09 95%CI 1.03–1.15; p = 0.001)) and COPD (OR 1.33, 95%CI 1.27–1.40; p < 0.001) demonstrate an increase in odds of a PCC diagnosis. Additionally, unvaccinated and partially vaccinated veterans exhibit significantly higher odds for PCC (p < 0.001) compared to fully vaccinated veterans in both the hospitalized and non-hospitalized cohorts. Increasing age, increasing BMI, female sex, Hispanic ethnicity, and veterans residing in the Southwestern United States show a significant (p < 0.05) increase in risk for a positive diagnosis of PCC in both groups.

**Conclusions:**

Veterans with pre-existing COPD or those hospitalized at the time of COVID-19 (indicating disease severity) are at higher risk of receiving a PCC diagnosis.

## Abstract (web summary)

Miller et al. examine the possible association between pre-existing comorbidities and hospitalization with subsequent diagnosis of post-COVID conditions (PCC) among Veterans. Veterans with pre-existing chronic obstructive pulmonary disease or hospitalization during their COVID-19 disease were more likely to be diagnosed with PCC.

## Plain language summary

Although most COVID-19 survivors return to their baseline health within two weeks, a large number of individuals continue experiencing symptoms, referred to as Long COVID. This study used electronic medical records from the US Department of Veterans Affairs, specifically records from September 1, 2021, to July 31, 2023, to study this disease. Our analysis revealed an increased risk for Long COVID diagnosis in veterans with chronic obstructive pulmonary disease (COPD) and those who hospitalized. Increasing age and body mass index, female sex, Hispanic ethnicity, and veterans residing in the Southwestern United States also showed significantly increased risk for contracting Long COVID. In conclusion, veterans with pre-existing COPD or those who were hospitalized at the time of COVID-19 (indicating disease severity) were more likely to be diagnosed with Long COVID.

## Introduction

In December 2019, the emergence of the Severe Acute Respiratory Syndrome Coronavirus 2 (SARS-CoV-2) infection marked the onset of the ongoing COVID-19 pandemic. Although a majority of individuals who survived COVID-19 manage to recover and return to their usual health within a span of two weeks, a notable proportion of individuals continue to grapple with an array of enduring health issues, collectively referred to as Post-COVID Conditions (PCC), also referred to as Long-COVID^1,2^. Estimates indicate that around 43% or more of those who contracted COVID-19 worldwide may have developed persistent PCC^3,4^. Notably, the identification and treatment of PCC/Long-COVID exhibits considerable variation, contingent on the specific criteria adopted and the population under study. The lack of a standardized definition has also led to underreporting of PCC cases^5–7^.

Even to date, the lack of consensus persists regarding the precise definition of PCC, with the Centers for Disease Control and Prevention (CDC) and the World Health Organization (WHO) putting forth differing interpretations. The CDC defines PCC as an infection-associated chronic condition that can occur after SARS-CoV-2 infection and is present for at least 3 months and affect one or more organ systems^8^. In contrast, the WHO defines PCC as the continuation or development of new symptoms 3 months after the initial SARS-CoV-2 infection, with these symptoms lasting for at least 2 months with no other explanation^9^. Within the Veterans Administration, PCC/Long-COVID is defined as symptoms or conditions that manifest or worsen at least four weeks subsequent to SARS-CoV-2 infection and is used when reporting PCC within the nationwide VA hospital systems.

Recognizing the prevalence of PCC, the WHO officially designated it as a distinct ailment and assigned the International Statistical Classification of Diseases and Related Health Problems, Tenth Revision (ICD-10) code U09.9, which became effective from October 1, 2021, and collectively define all unspecified Post-COVID Conditions^10^. Although medical practitioners have been employing this ICD-10 code following its approval, clear criteria for its use are yet to be solidified, which could result in some instances of PCC being misdiagnosed or undiagnosed. Among the frequently noted symptoms of PCC are general unease, persistent fatigue, difficulties in concentrating, shortness of breath, and a diminished quality of life^11^. Notably, it has been reported that the severity of PCC symptoms tends to be more pronounced among females, individuals of older age, those with weaker baseline health, and those who received more intensive care during their COVID-19 disease^12–17^. Veterans constitute one such group, as they often exhibit poorer baseline health compared to the general population^18^, and are expected to have a higher prevalence proportion than the general public.

Similar to the general population, limited published veterans’ data found associations between PCC and older age, female gender, race, documentation of high Charlson Comorbidity Index (CCI) score, vaccination status, and those who were hospitalized for initial COVID-19^1,19^. Moreover, based on overwhelming reports of persistent symptoms post-COVID-19, the Department of Veterans Health Administration has opened 22 multispecialty clinics across the US to provide optimal care to veterans with PCC^20^. Despite this response, the existing VA directive lacks information on the specific disease characteristics for defining the U09.9 ICD-10 code.

To gain a better understanding of risk factors associated with PCC in veterans, we hypothesized that hospitalization during COVID-19 and pre-existing comorbidities would increase the odds of PCC diagnosis. To attain these objectives, we conducted a retrospective cohort study using the Veterans Affairs (VA) COVID-19 Shared Data Resources with and without clinically diagnosed PCC. This manuscript investigated the demographic and health status attributes of US veterans hospitalized due to COVID-19 and then statistically analyzed the data using univariable and multivariable logistic regression models.

## Methods

### Data source

The study protocol underwent review by the Institutional Review Boards of both the University of Utah and the Department of VA Salt Lake City Hospital System. Following assessment, it was determined that informed consent was waived due to the use of deidentified data, and the protocol was exempt from further review. Ethical approvals (IRB# 00165075) were officially granted on May 1, 2023. The data for this study were sourced from the VA’s Corporate Data Warehouse (CDW) and the VA’s COVID-19 Shared Data Resources. All data were accessed and processed using the VA Informatics and Computing Infrastructure (VINCI) to ensure the preservation of veterans’ privacy and data security. The data for the present study was collected on September 5, 2023.

### Study design

In this retrospective cohort study, the focus was on veterans who were diagnosed with COVID-19 between September 1, 2021, and July 31, 2023, and survived at least 28 days. The study defined COVID-19 index dates as either the date of the first positive SARS-CoV-2 PCR or antigen test or, if veterans were already receiving care within 15 days before the positive test date, the date of hospital admission. The outcome was veterans who were subsequently diagnosed with PCC using ICD-10 Code U09.9 at least 28 days after their index date. The patient characteristics collected included demographic details, clinical attributes, reported pre-infection comorbidities, COVID-19 index date, whether or not more than one SARS-CoV-19 infection, types of treatment regimens during any COVID-19 episodes (if multiple infections were reported), PCC diagnosis date, and the vaccination status prior to the first COVID-19 index date. A COVID-19 diagnosis was determined through the use of the variable Ever Positive in the VA CDW. Ever Positive is defined as a positive PCR test, positive antigen test or evidence of positivity in clinical notes extracted by natural language processing. For patients with multiple COVID-19 diagnoses, multiple infections were not distinguished from reinfection.

The study employed two outcome groups for the statistical analyses: patients diagnosed with PCC (as indicated by the medical records of ICD-10 code U09.9), and those without this diagnosis. A hierarchical model was used to determine possible correlated variables (data not shown). It was determined that the COVID-19 treatments variables became insignificant upon inclusion of the hospitalized variable, and as such was removed from variable set before creating the multivariable models. Univariable and multivariable logistic regression models were employed to understand the potential associations between various variables and the assignment of the ICD-10 U09.9 code for PCC diagnosis. Patient comorbidities within 2 years prior to the index date were included. Both prevalence and incidence of PCC diagnosis were also computed.

#### Inclusion/exclusion criteria

**Fig. 1:**
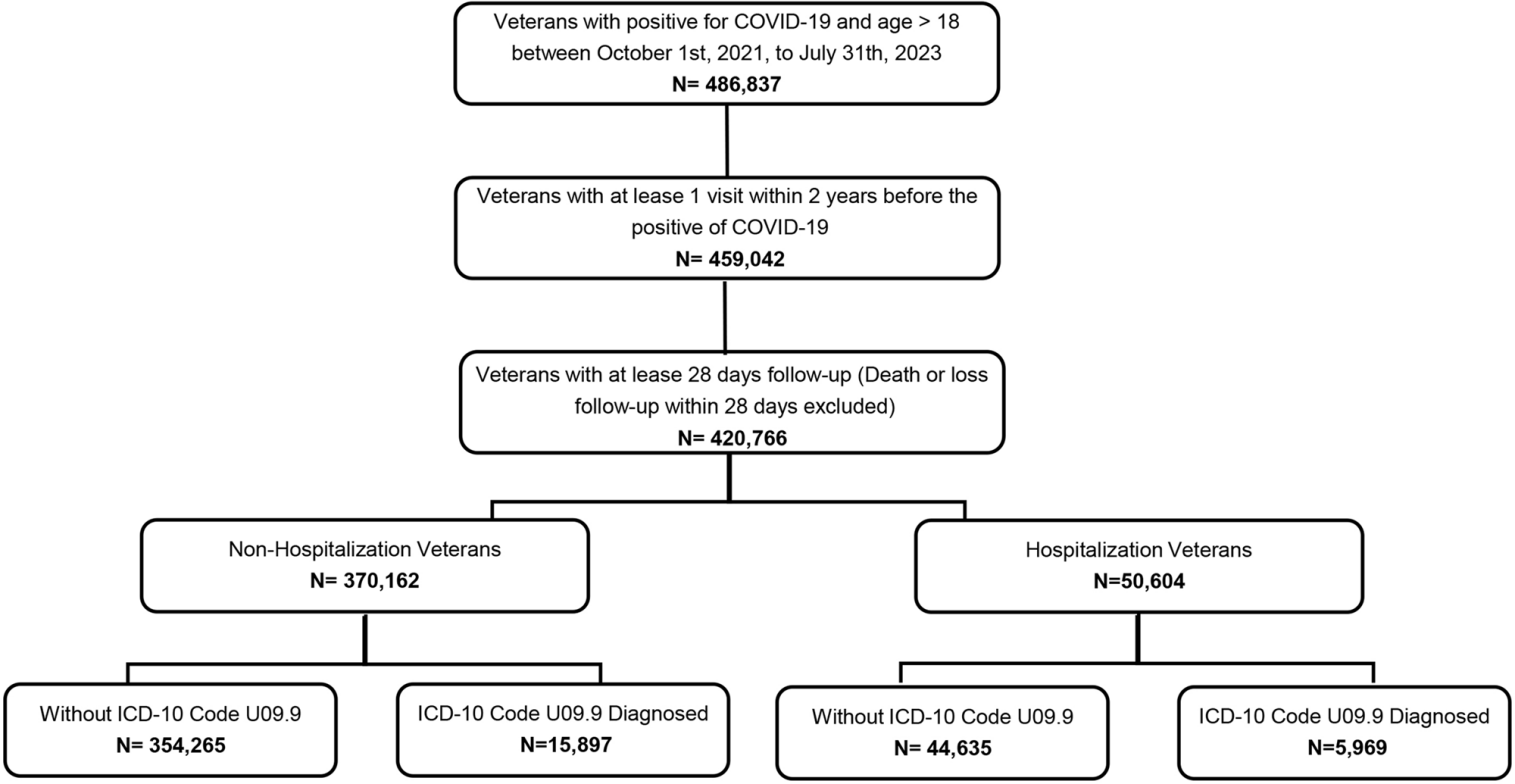
Study Cohort Selection. A flow chart displaying the study cohort selection.

Veterans who were 18 years or older, had documentation in the electronic health record as Ever Positive for SARS-CoV-2 infection between September 1, 2021, and July 31, 2023, had at least 1 clinical visit within the 2 years prior to a COVID-19 positive diagnosis and survived over 28 days were included. Those who perished within 28 days post-SARS-CoV-2 infection were excluded from this study. Also, there was no limitation placed on the number of times PCC episodes were reported, but a unique patient ID was only counted once. A flow chart of the cohort selection process is given in Fig. 1.

#### Statistical and reproducibility

The monthly incidence proportion was calculated as the number of new reported PCC diagnoses during the month divided by accumulative SARS-CoV-2 infected veterans who were still at risk for PCC (i.e., they had yet been diagnosed with the PCC). The prevalence proportion was calculated as the sum of all PCC diagnoses up to and including that month, divided by the total number of veterans who were previously diagnosed with SARS-CoV-2 infections and survived.

As previously mentioned, pertinent clinical and demographic characteristics were extracted from veterans’ electronic medical records. This encompassed variables such as age, gender, race/ethnicity, comorbidities, severity of the acute illness, hospitalization status, and treatments administered. The descriptive analysis was conducted on all eligible COVID-19 positive veterans, both those with and without the ICD-10 U09.9 diagnosis. To facilitate analysis, categorical variables were established for age, sex (male or female), race (White, Black, Asian, Native American, and other), ethnicity (Hispanic or Latino, NOT Hispanic or Latino, Unknown), geographical regions (West, Midwest, Northwest, Southwest, Southeast, and others), levels of rurality (city, rural, urban), BMI and vaccination status (never, partially, fully vaccinated) at the time of SARS-CoV-2 diagnosis. Fully vaccinated was defined as having received the recommended doses for each vaccine type. Moreover, for illnesses known to exacerbate the severity of COVID-19, dichotomous variables were established based on the 24-month first pre-index date electronic records. All patients lacking at least one VA visit 24-month prior to their index date were removed from the study cohort. Treatments administered during the SARS-CoV-2 infection included whether patients were hospitalized, required mechanical ventilation, received oxygen therapy, or were treated with antibiotics, antiviral medications, and corticosteroids within 60 days of the index date. The data were presented as percentages (%) for categorical variables.

Univariable logistic regression models were employed initially to assess the significance of the relationships between specific variables and the ICD-10 U09.9 diagnostic code reports. Subsequently, multivariable logistic regression models were utilized to examine the association between covariates and PCC diagnosis. For this study, the multivariable models were adjusted for all clinical and demographic characteristics that were examined in the univariable logistic regression analyses. As the PCC diagnosis incidence did not exceed 2.41%, which is less than 10% and so meets the rare disease assumption^21^, the odds ratio (OR) can be interpreted as a relative risk. All P-value measurements are two-sided.

The data analyses were conducted using the STATA version 17 software package (StataCorp LLC, College Station, Texas).

### Reporting summary

Further information on research design is available in the Nature Portfolio Reporting Summary linked to this article.

## Results

### PCC Incidence and Prevalence

**Fig. 2:**
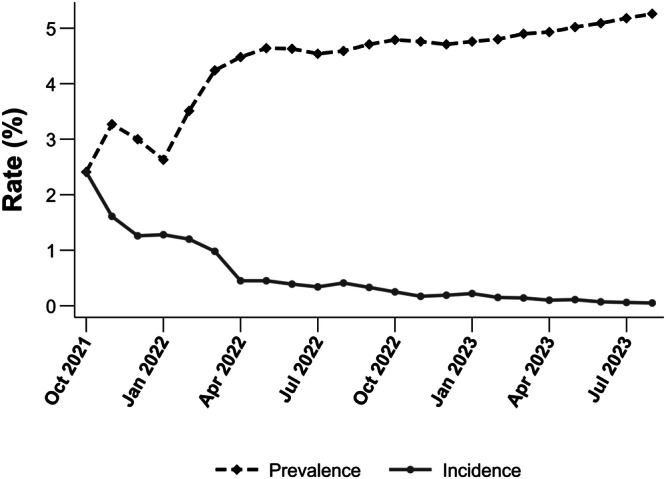
Prevalence and Incidence Rates of PCC Diagnosed Veterans. Line graphs showing the monthly prevalence and incidence of PCC diagnoses within the veteran population. The initial variations plateaued after April 2022, perhaps related to variabilities of recording ICD 10 code or waning efficacy of immunity following initial vaccination^64^.

There were 514,328 U.S. veterans older than 18 years of age, and diagnosed with COVID-19 within the study period. Of those, 23,496 individuals (5.3%) were diagnosed with PCC (Fig. 1). On average, veterans were diagnosed with PCC 73 days (mean), with a median of 34 (IQR 14–77) days, after the initial diagnosis of SARS-CoV-2 infection. At least 50% of veterans were diagnosed with this disease within 34 days after the reported index date. Figure 2 displays the monthly incidence and prevalence, revealing an upward trajectory for prevalence during the initial stages that eventually plateaued at around 4.48 by April 2022. The monthly incidence fluctuated between 2.41 and 0.05%.

### Demographics

The characteristics of veterans with electronic records of PCC diagnosis and those without were summarized for comparison in Table 1, which provides the demographic characteristics of the entire veteran cohort, comprising individuals with one or more instances of COVID-19 diagnosis. A similar table containing the same characteristics was also prepared consisting of veterans with and without a COVID-19 diagnosis (Supplementary Table 1). Veterans with and without PCC diagnosis were predominantly White (71.9, 65.0%), male (86.3, 84.6%), and 71–80 years old (28.1, 24.6%, respectively). In both groups, Veterans BMI ≥ 30 (48.5, 45.0%) was the most predominant weight category (Table 2).

**Table 1:** Demographic Characteristics of Veterans Diagnosed with SARS-CoV-2 Infection, Further Subdivided by PCC Diagnosis

	Total	PCC Negative	PCC Positive	p-value
	N = 444,991	N = 421,495	N = 23,496	
**Age (Year)**				<0.001
18 to 40	73,036 (16.4%)	70,381 (16.7%)	2655 (11.3%)	
41 to 50	55,506 (12.5%)	52,725 (12.5%)	2781 (11.8%)	
51 to 60	80,336 (18.1%)	76,218 (18.1%)	4118 (17.5%)	
61 to 70	91,947 (20.7%)	86,981 (20.6%)	4966 (21.1%)	
71 to 80	110,322 (24.8%)	103,727 (24.6%)	6595 (28.1%)	
≥81	33,844 (7.6%)	31,463 (7.5%)	2381 (10.1%)	
**Gender**				<0.001
Male	376,657 (84.6%)	356,377 (84.6%)	20,280 (86.3%)	
Female	68,334 (15.4%)	65,118 (15.4%)	3216 (13.7%)	
**Race**				<0.001
White	290,682 (65.3%)	273,785 (65.0%)	16,897 (71.9%)	
American Indian or Alaska Native	3572 (0.8%)	3336 (0.8%)	236 (1.0%)	
Asian	5934 (1.3%)	5680 (1.3%)	254 (1.1%)	
Black or African American	88,918 (20.0%)	85,392 (20.3%)	3526 (15.0%)	
Native Hawaiian or Other Pacific Islander	4286 (1.0%)	4039 (1.0%)	247 (1.1%)	
Unknown	51,599 (11.6%)	49,263 (11.7%)	2336 (9.9%)	
**Ethnicity**				<0.001
Hispanic or Latino	39,222 (8.8%)	35,813 (8.5%)	3409 (14.5%)	
Not Hispanic or Latino	365,843 (82.2%)	347,150 (82.4%)	18,693 (79.6%)	
Unknown	39,926 (9.0%)	38,532 (9.1%)	1394 (5.9%)	
**Region**				<0.001
West	67,916 (15.3%)	64,904 (15.4%)	3012 (12.8%)	
Midwest	77,775 (17.5%)	74,074 (17.6%)	3701 (15.8%)	
Northeast	56,889 (12.8%)	54,686 (13.0%)	2203 (9.4%)	
Southeast	120,702 (27.1%)	116,432 (27.6%)	4270 (18.2%)	
Southwest	47,146 (10.6%)	40,944 (9.7%)	6202 (26.4%)	
Others/Unknown	74,563 (16.8%)	70,455 (16.7%)	4108 (17.5%)	
**Rurality**				<0.001
City Town	32,123 (7.2%)	30,319 (7.2%)	1804 (7.7%)	
Small Town Rural	24,366 (5.5%)	23,021 (5.5%)	1345 (5.7%)	
Urban	320,228 (72.0%)	303,750 (72.1%)	16,478 (70.1%)	
Unknown	68,274 (15.3%)	64,405 (15.3%)	3869 (16.5%)	

Demographic characteristics of veterans with SARS-CoV-2 infection between September 2021 and July 2023 were subsequently categorized based on the presence or absence of ICD-10 U09.9 in their medical records. On average, 68.8 days after the index day of the COVID-19, the ICD-10 code was posted. All section headings are bolded. *PCC* Post-COVID Conditions. Unknown: missing data.

**Table 2:** Additional Demographic Characteristics of Veterans Diagnosed with SARS-CoV-2 Infection, Further Subdivided by PCC Diagnosis

	Total	PCC Negative	PCC Positive	p-value
	N = 444,991	N = 421,495	N = 23,496	
**BMI groups(kg/m**^**2**^**)**				<0.001
Underweight (<18.5)	5306 (1.2%)	4953 (1.2%)	353 (1.5%)	
Normal weight (18.5–24.9)	78,672 (17.7%)	74,512 (17.7%)	4160 (17.7%)	
Overweight (25–91.9)	145,029 (32.6%)	137,593 (32.6%)	7436 (31.6%)	
Obese (30–39.9)	170,914 (38.4%)	161,451 (38.3%)	9463 (40.3%)	
Morbidly Obese (40 + )	30,121 (6.8%)	28,198 (6.7%)	1923 (8.2%)	
Unknown	14,949 (3.4%)	14,788 (3.5%)	161 (0.7%)	
**Smoking Status**				<0.001
Current Smoker	68,441 (15.4%)	65,358 (15.5%)	3083 (13.1%)	
Former Smoker	165,105 (37.1%)	155,521 (36.9%)	9584 (40.8%)	
Never Smoked	167,338 (37.6%)	157,841 (37.4%)	9497 (40.4%)	
Unknown	44,107 (9.9%)	42,775 (10.1%)	1332 (5.7%)	
***Comorbidities (within 2 years pre-index date)***				
Chronical Kidney Disease (CKD)	57,282 (12.9%)	53,339 (12.7%)	3943 (16.8%)	<0.001
Liver disease	32,323 (7.3%)	30,333 (7.2%)	1990 (8.5%)	<0.001
Hypertension	255,704 (57.5%)	240,723 (57.1%)	14,981 (63.8%)	<0.001
Diabetes	134,396 (30.2%)	126,103 (29.9%)	8293 (35.3%)	<0.001
COPD	65,116 (14.6%)	60,449 (14.3%)	4667 (19.9%)	<0.001
**COVID-19 positivity record**				
COVID-19 disease more than once	23,893 (5.4%)	23,103 (5.5%)	790 (3.4%)	<0.001
**Vaccination status**				<0.001
Full Vaccinated	294,168 (66.1%)	279,378 (66.3%)	14,790 (62.9%)	
Partially vaccinated	23,806 (5.3%)	22,104 (5.2%)	1702 (7.2%)	
Unvaccinated	127,017 (28.5%)	120,013 (28.5%)	7004 (29.8%)	
**Hospitalized**				
Hospitalization or ICU	53,663 (12.1%)	47,153 (11.2%)	6510 (27.7%)	<0.001
**Treatment for COVID-19**				
Mechanical Ventilation use	11,557 (2.6%)	9617 (2.3%)	1940 (8.3%)	<0.001
Oxygen Therapy	30,157 (6.8%)	25,344 (6.0%)	4813 (20.5%)	<0.001
Antibiotic meds use	69,405 (15.6%)	62,680 (14.9%)	6725 (28.6%)	<0.001
Antiviral meds use	73,521 (16.5%)	67,507 (16.0%)	6014 (25.6%)	<0.001
Corticosteroid use	76,617 (17.2%)	69,250 (16.4%)	7367 (31.4%)	<0.001

Selected clinical characteristics of veterans with SARS-CoV-2 infection between September 2021 and July 2023 and were subsequently categorized based on the presence or absence of ICD-10 U09.9 code in their medical records. All section headings are bolded. *PCC*: Post-COVID Conditions. Unknown: missing data.

### Clinical characteristics

Table 2 shows selected pertinent health characteristics, which shows the proportions of veterans displaying comorbidities, smoking habits, vaccination status, and the medical treatments that veterans underwent during their SARS-CoV-2 infection(s). Within the PCC diagnosis outcome group, Former Smoker was the most common smoking status (40.8%), while Never Smoked was the most common (40.4%) among the group not diagnosed with PCC. The majority of veterans (63.8%) were reported to have hypertension. Additionally, veterans with PCC had a higher prevalence of chronic kidney disease (CDK), liver diseases, diabetes, and chronic obstructive pulmonary disease (COPD) compared to veterans without a PCC diagnosis. A lower proportion of veterans (3.4%) diagnosed with PCC had experienced more than one SARS-CoV-2 infection episode (i.e., two U09.9 diagnoses greater than 28 days apart from each other). Among those in the PCC diagnosis outcome group, a higher proportion had been hospitalized (27.7 vs. 11.2%), required ventilation (8.3 vs. 2.3%), received oxygen therapy (20.5 vs. 6.0%), or were administered antibiotics (28.6 vs. 14.9%), antiviral medications (25.6 vs. 16.0%), or steroids (31.4 vs. 16.4%). However, the vaccination statuses of the veterans were similar between the two groups.

### Logistic regression models

**Fig. 3:**
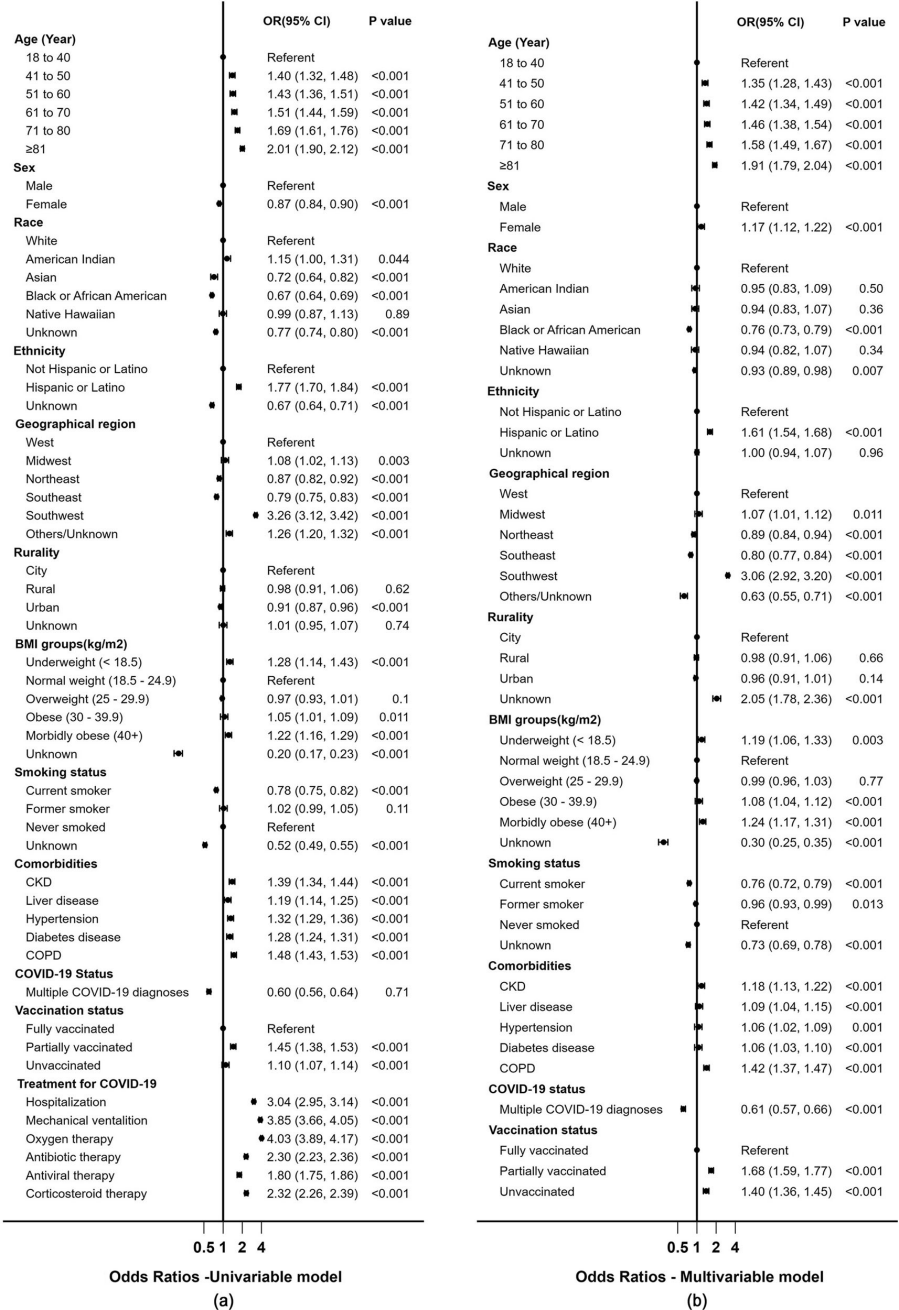
Odds Ratios for Characteristics Associated with PCC Diagnosis in Veterans. Forest plots of univariable **a** and multivariable **b** logistic regression models illustrating the relationships between demographics, health factors, and hospitalization and their association with PCC diagnosis in clinically confirmed cases among veterans. CKD Chronic Kidney Disease, COPD Chronic Obstructive. ‘Unknown’ refers to those without any entry for the respective categories. N = 444,991 patients.

As depicted in Fig. 3, logistic regression models were then employed to compute the ORs. Initial univariable analyses indicated that compared to individuals aged 18–40, older veterans had relatively higher odds of a PCC diagnosis (a relative increase in risk ranging from 40 to 101%). Additionally, sex, race, ethnicity, geographical location, rurality, BMI, smoking status, pre-existing comorbidities, vaccination status, hospitalization, and subsequent treatments for COVID-19 demonstrated significant associations with subsequent diagnosis of PCC diagnosis (Fig. 3, Left). These categories were adjusted in the multivariable model (Fig. 3, Right).

The adjusted ORs indicated that increasing age (35–91%), female (17%), Hispanic ethnicity (61%), veterans residing in the southwest US region (206%), BMI of 40+ (24%), COPD (42%) and being not fully vaccinated (40–68%) increased the significant relative risks for PCC diagnosis compared to their respective referents. In contrast, African Americans were 24% (adjusted OR 0.76, 95%CI 0.73–0.79; p < 0.001) less likely to be diagnosed with this disease compared to the White veteran referent. Notably, increasing age and increasing BMI remained significant within the adjusted model. Particularly striking was the finding that veterans from the Southwest region of the USA had approximately three times increased relative odds for PCC diagnosis compared to those from the West.

**Fig. 4:**
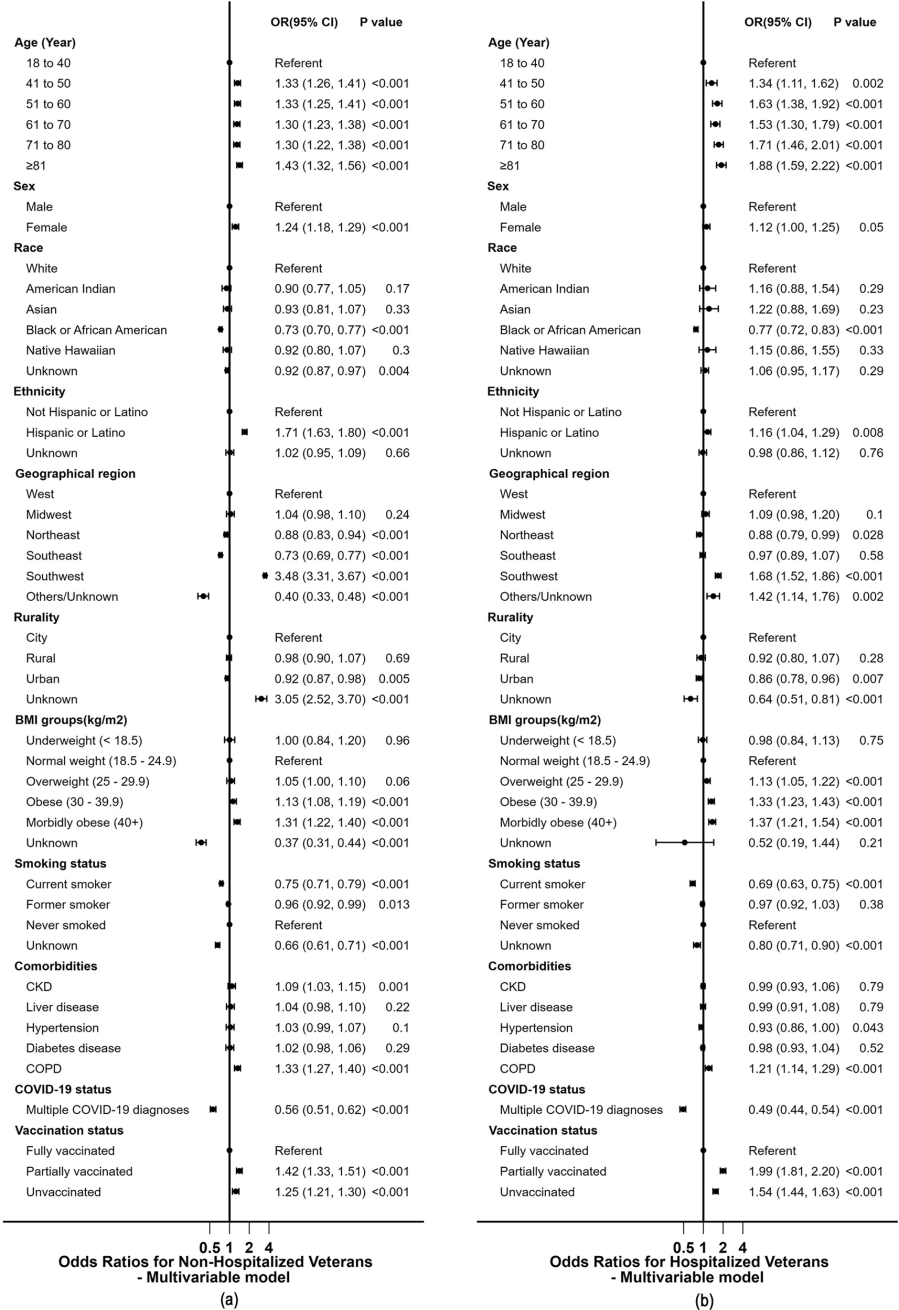
Odds Ratios for Characteristics Associated with PCC Diagnosis for Non-Hospitalized and Hospitalized Veterans. Forest plots showing multivariable logistic regression models predicting the PCC diagnoses outcome in veterans. Hospitalization was used as the surrogate for COVID-19 severity. **a** (N = 391,328 patients) Those veterans who, during their COVID-19 disease, were hospitalized (severe cases) and **b** (N = 53,663 patients) those without hospitalization (less severe cases). CKD Chronic Kidney Disease, COPD Chronic Obstructive Pulmonary Disease. ‘Unknown’ refers to those without any entry for the respective categories.

Given that the adjusted model now predicts hospitalization for COVID-19 as a risk factor for subsequent diagnosis of PCC, we conducted multivariable subgroup analyses on both non-hospitalized and hospitalized veterans (see Fig. 4). In terms of comorbidities, while liver disease, hypertension, and diabetes no longer exhibited significance for increased risk of PCC in the non-hospitalized group, CKD and COPD maintained significant associations with PCC. Likewise, within the hospitalized cohort, only COPD showed a significant 21% increased relative odds for PCC diagnosis. Notably, veterans who were unvaccinated during the COVID-19 illness stage, older adults, those residing in the Southwest U.S., and those with higher BMI had increased relative odds within the hospitalized cohort when compared to those who did not. Black veterans’ adjusted odd ratios revealed that they sustained their relatively protective risk association with PCC in both subgroups.

## Discussion

As stated in the introduction, this study tested the hypothesis that hospitalization for the COVID-19 and pre-existing comorbidities would increase the odds of PCC. The data supported the hypothesis, showing hospitalization and pre-existing comorbidities increased the relative odds of PCC diagnosis. Interestingly, the unvaccinated veterans showed significantly increased relative risk for PCC diagnosis. Also, multiple COVID-19 episodes showed a reduced risk for PCC diagnosis in both non-hospitalized and hospitalized veterans, and it is in agreement with literature^22^. Among the veteran population receiving care through the nationwide VA hospital systems, subsequent to the introduction of the ICD-10 U09.9 diagnosis code, the monthly prevalence of PCC diagnosis exhibited a range from 2.0 to 4.5 % (at the commencement and culmination of the study period, respectively). The incidence proportion varied from 2.41 to 0.05%. These proportions met the rare disease assumption^21^; thus, the reported ORs were interpreted as relative risks. On average, PCC diagnosis was documented 73 days, 34 (IQR, 14–77) median days, after the SARS-CoV-2 infection index day, supporting published VA data^23^.

It is worth noting that many symptoms, such as fatigue, deteriorated quality of life, dyspnea, joint pain, chest discomfort, cough, skin rashes, palpitations, headaches, diarrhea, and sensations like ‘pins and needles,’ along with anxiety, depression, and post-traumatic stress disorder, are reported with PCC^24–31^. Although the prevalence and incidence of each individual symptom among veterans diagnosed with PCC may vary, we did not attempt to calculate them individually. The veterans’ data from EHR indicated that, overall, a lower prevalence of PCC diagnosis (5.3%) compared to what has been reported by the CDC (6.0–11%)^32^ and other studies^33–35^. These differences were not to be expected within the veteran population, given their free access to the healthcare system. These rate differences could also be attributed to the study design and reporting bias. Study designs that prospectively follow patients or use surveys can be expected to measure higher incidence and prevalence than patient medical records that rely on the ICD-10 code U09.9, as with this study design.

Veterans, relative to non-veterans, tend to have multiple chronic conditions^36^. Nittas and colleagues reported prevalence estimates ranging from 7.5 to 41.0% based on an umbrella review and a targeted evidence synthesis of 102 studies^34^. Another meta-analysis reviewing 63 studies noted high heterogeneity in prevalence reporting across different follow-up periods^33^. Raveendran et al. reported a higher prevalence of approximately 87% among hospitalized patients for residual symptoms compared to those treated for COVID-19 on an outpatient basis^15^. Although we have yet to attempt to differentiate between prevalence among hospitalized and non-hospitalized cohorts, these differences may be attributed to the complexity and lack of clarity in diagnosing and reporting the PCC diagnosis. The variability in prevalence data can be attributed to differences in study periods (pre- or post- ICD-10 code availability), symptom definitions (CDC vs. WHO), study designs, and reporting practices. Since the introduction of the ICD-10 code for PCC diagnosis, our data (Fig. 2) clearly indicated that from April 2022, the prevalence has stabilized at around 4.5% with new incidence rates of 0.5%, suggesting increasing clarity with reporting of the ICD-10 code and/or veterans did not seek help unless it was physically debilitating.

Most importantly, our data also aligned with prior research indicating that veterans who were hospitalized for acute COVID-19 exhibit a relative 41% increased risk of having a PCC diagnosis on their health record compared to non-hospitalized veterans^37–39^. The PCC may have been driven by extended tissue damage during the severe infection period, affecting organs such as the pulmonary, muscular, neural, and cardiac, as well as pathological systemic inflammation. Recent articles suggested that there were differences in exaggerated humoral responses relative to matched controls in a cross-sectional study^40^. Again, our data suggest that COVID-19 severity may be related to the subsequent presentation of PCC^40–42^.

The presented data demonstrated significantly increased relative risk (p < 0.05) associations with CKD and COPD, and PCC diagnosis in non-hospitalized veterans. However, within the hospitalized cohort, only COPD was associated with increased risk (p < 0.001). As found in the literature, the presence of diabetes is expected to influence the exacerbation of persisting residual symptoms of COVID-19 via various pathophysiological mechanisms, but our analysis did not find an association with PCC diagnosis. Moreover, it has been reported in a prospective study that included 108 patients with Type 2 diabetes who had COVID-19, as compared to those without, had significantly more fatigue after the acute illness^35^, which may be inadequately powered compared to the data presented here. It is also worth mentioning that the above study^35^ used subjective scores such as patient-reported assessment of their fatigue, and their statistical analysis was also limited to reporting group differences^35^.

A few studies have consistently suggested that PCC diagnosis is more prevalent in females^26,43,44^ than their male counterparts. Our data corroborates this trend, indicating that non-hospitalized female veterans had a relative 24% higher odds of having a PCC diagnosis compared to male veterans, which was slightly lower (12%) in the hospitalized group; though it was insignificant (p = 0.05). While many studies have reported a two- to four-fold increased risk for female patients^43,45^, our findings suggest a more modest increased risk. This discrepancy could potentially be attributed to the sample bias inherent to the veteran dataset, which predominantly consists of males (approximately 85%; Table 1). Although gender disparities have been evident in COVID-19 data^46,47^, with males exhibiting higher mortality rates during COVID-19, the existing literature has not provided a definitive explanation for why PCC diagnosis disproportionately affects females. This disparity may be linked to the inherent immunological differences between sexes or hormonal factors. It is established that females generally mount a stronger immune response to infections^48–51^, which could result in a prolonged immune reaction in some cases, potentially elevating the risk of receiving PCC diagnosis. Additionally, hormonal variations between males and females, including the presence of estrogen and progesterone, can influence immune responses and inflammatory processes^52^. These hormonal distinctions may contribute to the development of PCC diagnosis, necessitating further research to elucidate their precise role in this phenomenon.

While our adjusted odds ratio indicates a relative 35–85% increased risk among individuals aged 41 and above (Fig. 3, Right), the broader literature presents conflicting findings regarding the association between age and PCC diagnosis^19,26,44,53^. For instance, Notarte and colleagues analyzed 38 studies and concluded that age is not linked to the presentation of PCC^44^, while another systematic review identified older age as a risk factor^26^. Interestingly, within the subset of literature that employed U09.9 chart records to identify PCC cases, adjusted odds ratios did demonstrate an association between age and PCC diagnosis^19^, supporting our finding. Farmer et al. also found that, in general, veterans who seek health care within the VHA system are generally older and sicker than other veterans who received healthcare elsewhere^54^, possibly contributing to the disparities in risk reporting.

Our adjusted overall odds ratios (Fig. 3) revealed a relative 8 and 24% increased risks for PCC diagnosis among obese and morbidly obese veterans, respectively. This is in agreement with existing research findings from several sources^55–58^. It is important to underscore that both obesity and veterans’ overall health have been established as substantial risk factors for the severity of COVID-19^59^ and the subsequent diagnosis of PCC^57^, thus validating our data. This association could be attributed to the persistent inflammation linked to obesity and the modified immune responses during COVID-19, which contribute to severe illness and serve as risk factors for the emergence of long-term COVID-19 complications (PCC)^60–62^. Consistent with prior research, our veteran-focused data revealed a lower risk of having a PCC diagnosis in the Black race (OR 0.73 for non-hospitalized and OR 0.77 for hospitalized veterans) compared to White counterparts, indicating a degree of protection. This may also be indicative of the underrepresentation of minority races within the study cohort, suggesting underdiagnosis of this group of individuals. When comparing non-Hispanics, there was an elevated relative risk of having a PCC diagnosis in the Hispanic/Latino ethnicity (refer to Fig. 3) in both non-hospitalized and hospitalized cohort. Findings from another veterans’ study, which employed an ICD-10 code to identify PCC cases, corroborated our results^19^. Of particular significance, there was an over 3-fold increase in the odds of PCC diagnoses among veterans in the Southwestern U.S. region, where most of the population identified as of Hispanic origin. This observation highlights a disproportionate prevalence of PCC diagnosis within the Hispanic community, further supporting CDC data^63^.

This study comes with certain limitations. First, our data only captures diagnoses documented in the electronic medical records. Consequently, PCC may be underdiagnosed among individuals who were less likely to seek medical care for COVID-19 or PCC either due to mild disease presentation or non-clinical factors such as distance to a healthcare facility. Thus, prevalence and incidence rates may have been underreported. Additionally, the methods used in this manuscript to calculate the incidence and prevalence use the assumption that patients do not recover from PCC. Given that this study is retrospective in nature, it is advisable to conduct a prospective study to comprehensively understand the associations between hospitalization and treatment with the diagnosis of PCC. Furthermore, to investigate the potential association between vaccination and PCC diagnosis, it is essential to confirm these findings through a prospective controlled or propensity-matched study, especially since vaccination provides protection for a limited duration. Additionally, considering that long-term COVID symptoms are diverse and have not been explored, it may be valuable to survey individual symptoms as well, which was not investigated in this study.

## Supplementary information

Supplementary Data 1

Supplemental Table 1

Reporting Summary

## Data Availability

Source data for Fig. 2 is in Supplementary Data 1. The other source data that support the findings of this study are available from the US Department of Veterans Affairs. VA data are made freely available to researchers behind the VA firewall with an approved VA study protocol. For more information on obtaining an approved VA study protocol, visit https://www.virec.research.va.gov or contact the VA Information Resource Center at VIReC@va.gov.
