# Supplementary material for: Pre-existing comorbidities and hospitalization for COVID-19 are associated with post-COVID conditions in the U.S. veteran population
Source: Commun Med (Lond). 2025 Oct 24;5:442. doi: 10.1038/s43856-025-01057-5 (PMC12552489; doi:10.1038/s43856-025-01057-5)
Supplement: Supplementary file 2 — Supplemental Table 1 [file 43856_2025_1057_MOESM2_ESM.docx]

***Supplementary Table 1 Title:*** Demographic Characteristics of Veterans Diagnosed with and without SARS-CoV-2 Infection via the Diagnostic Code U07.1

|  | **Total** | **U07.1 Negative** | **U07.1 Positive** | **p-value** |
| --- | --- | --- | --- | --- |
|  | **N=23,496** | **N=12,879** | **N=10,617** |  |
| **Age (Year)** |  |  |  | <0.001 |
| 18 to 40 | 2,655 (11.3%) | 1,656 (12.9%) | 999 ( 9.4%) |  |
| 41 to 50 | 2,781 (11.8%) | 1,687 (13.1%) | 1,094 (10.3%) |  |
| 51 to 60 | 4,118 (17.5%) | 2,339 (18.2%) | 1,779 (16.8%) |  |
| 61 to 70 | 4,966 (21.1%) | 2,716 (21.1%) | 2,250 (21.2%) |  |
| 71 to 80 | 6,595 (28.1%) | 3,398 (26.4%) | 3,197 (30.1%) |  |
| ≥81 | 2,381 (10.1%) | 1,083 ( 8.4%) | 1,298 (12.2%) |  |
| **Gender** |  |  |  | <0.001 |
| Female | 20,280 (86.3%) | 11,023 (85.6%) | 9,257 (87.2%) |  |
| Male | 3,216 (13.7%) | 1,856 (14.4%) | 1,360 (12.8%) |  |
| **Race** |  |  |  | <0.001 |
| White | 16,897 (71.9%) | 9,356 (72.6%) | 7,541 (71.0%) |  |
| American Indian or Alaska Native | 236 ( 1.0%) | 142 ( 1.1%) | 94 ( 0.9%) |  |
| Asian | 254 ( 1.1%) | 149 ( 1.2%) | 105 ( 1.0%) |  |
| Black or African American | 3,526 (15.0%) | 1,745 (13.5%) | 1,781 (16.8%) |  |
| Native Hawaiian or Other Pacific Islander | 247 ( 1.1%) | 128 ( 1.0%) | 119 ( 1.1%) |  |
| Unknown | 2,336 ( 9.9%) | 1,359 (10.6%) | 977 ( 9.2%) |  |
| **Ethnicity** |  |  |  | <0.001 |
| Hispanic or Latino | 3,409 (14.5%) | 2,009 (15.6%) | 1,400 (13.2%) |  |
| Not Hispanic or Latino | 18,693 (79.6%) | 10,024 (77.8%) | 8,669 (81.7%) |  |
| Unknown | 1,394 ( 5.9%) | 846 ( 6.6%) | 548 ( 5.2%) |  |
| **Region** |  |  |  | <0.001 |
| West | 3,012 (12.8%) | 1,635 (12.7%) | 1,377 (13.0%) |  |
| Midwest | 3,701 (15.8%) | 2,029 (15.8%) | 1,672 (15.7%) |  |
| Northeast | 2,203 ( 9.4%) | 1,106 ( 8.6%) | 1,097 (10.3%) |  |
| Southeast | 4,270 (18.2%) | 2,159 (16.8%) | 2,111 (19.9%) |  |
| Southwest | 6,202 (26.4%) | 3,672 (28.5%) | 2,530 (23.8%) |  |
| Others/Unknown | 4,108 (17.5%) | 2,278 (17.7%) | 1,830 (17.2%) |  |
| **Rurality** |  |  |  | <0.001 |
| City Town | 1,804 ( 7.7%) | 1,074 ( 8.3%) | 730 ( 6.9%) |  |
| Small Town Rural | 1,345 ( 5.7%) | 801 ( 6.2%) | 544 ( 5.1%) |  |
| Urban | 16,478 (70.1%) | 8,823 (68.5%) | 7,655 (72.1%) |  |
| Unknown | 3,869 (16.5%) | 2,181 (16.9%) | 1,688 (15.9%) |  |
| **BMI groups(kg/m^2^)** |  |  |  | <0.001 |
| Underweight (< 18.5) | 353 ( 1.5%) | 173 ( 1.3%) | 180 ( 1.7%) |  |
| Normal weight (18.5 - 24.9) | 4,160 (17.7%) | 2,108 (16.4%) | 2,052 (19.3%) |  |
| Overweight (25 - 91.9) | 7,436 (31.6%) | 4,087 (31.7%) | 3,349 (31.5%) |  |
| Obese (30 - 39.9) | 9,463 (40.3%) | 5,354 (41.6%) | 4,109 (38.7%) |  |
| Morbidly Obese (40+) | 1,923 ( 8.2%) | 1,039 ( 8.1%) | 884 ( 8.3%) |  |
| Unknown | 161 ( 0.7%) | 118 ( 0.9%) | 43 ( 0.4%) |  |
| **Smoking Status** |  |  |  | 0.022 |
| Current Smoker | 3,083 (13.1%) | 1,759 (13.7%) | 1,324 (12.5%) |  |
| Former Smoker | 9,584 (40.8%) | 5,208 (40.4%) | 4,376 (41.2%) |  |
| Never Smoker | 9,497 (40.4%) | 5,159 (40.1%) | 4,338 (40.9%) |  |
| Unknown | 1,332 ( 5.7%) | 753 ( 5.8%) | 579 ( 5.5%) |  |
| **Comorbidities (within 2 years pre-index date)** |  |  |  |  |
| Chronical Kidney Disease (CKD) | 3,943 (16.8%) | 1,913 (14.9%) | 2,030 (19.1%) | <0.001 |
| Liver disease | 1,990 ( 8.5%) | 1,012 ( 7.9%) | 978 ( 9.2%) | <0.001 |
| Hypertension | 14,981 (63.8%) | 7,852 (61.0%) | 7,129 (67.1%) | <0.001 |
| Diabetes | 8,293 (35.3%) | 4,320 (33.5%) | 3,973 (37.4%) | <0.001 |
| COPD | 4,667 (19.9%) | 2,238 (17.4%) | 2,429 (22.9%) | <0.001 |
| **COVID-19 positivity record** |  |  |  |  |
| COVID-19 disease more than once | 790 ( 3.4%) | 380 ( 3.0%) | 410 ( 3.9%) | <0.001 |
| **Vaccination status** |  |  |  | <0.001 |
| Full Vaccinated | 14,790 (62.9%) | 7,995 (62.1%) | 6,795 (64.0%) |  |
| Partially vaccinated | 1,702 ( 7.2%) | 901 ( 7.0%) | 801 ( 7.5%) |  |
| Unvaccinated | 7,004 (29.8%) | 3,983 (30.9%) | 3,021 (28.5%) |  |
| **Hospitalized** |  |  |  |  |
| Hospitalization or ICU | 6,510 (27.7%) | 2,318 (18.0%) | 4,192 (39.5%) | <0.001 |
| **Treatment for COVID-19** |  |  |  |  |
| Mechanical Ventilation use | 1,940 ( 8.3%) | 727 ( 5.6%) | 1,213 (11.4%) | <0.001 |
| Oxygen Therapy | 4,813 (20.5%) | 1,615 (12.5%) | 3,198 (30.1%) | <0.001 |
| Antibiotic meds use | 6,725 (28.6%) | 2,870 (22.3%) | 3,855 (36.3%) | <0.001 |
| Antiviral meds use | 6,014 (25.6%) | 1,728 (13.4%) | 4,286 (40.4%) | <0.001 |
| Corticosteroid use | 7,367 (31.4%) | 3,070 (23.8%) | 4,297 (40.5%) | <0.001 |
|  | | | | |

***Supplementary Table 1 Legend:*** *Selected clinical characteristics of veterans with and without a SARS-CoV-2 infection, using the diagnostic code U07.1, between September 2021 and July 2023. All section headings are bolded. PCC: Post-COVID Conditions. Unknown: missing data.*
